# The Suitability of Dried Blood Spot Sampling for Pharmacokinetic Studies in Veterinary Medicine

**DOI:** 10.3390/vetsci12050488

**Published:** 2025-05-18

**Authors:** Anisa Bardhi, Andrea Barbarossa, Andrè Joubert, Ronette Gehring, Carlotta Lambertini, Noemi Romagnoli

**Affiliations:** 1 Department of Veterinary Medical Sciences, University of Bologna, 40064 Ozzano dell’Emilia (Bo), Italy; anisa.bardhi@unibo.it (A.B.); andrea.barbarossa@unibo.it (A.B.); noemi.romagnoli@unibo.it (N.R.); 2 Health Sciences and Technologies-Interdepartmental Centre for Industrial Research (CIRI-SDV), University of Bologna, 40064 Ozzano dell’Emilia (Bo), Italy; 3 Division of Veterinary Pharmacology and Pharmacy, Department of Population Health Sciences, Faculty of Veterinary Medicine, Utrecht University, 3584 CM Utrecht, The Netherlands; a.j.joubert@uu.nl (A.J.); r.gehring@uu.nl (R.G.)

**Keywords:** microsampling, research, medetomidine, ketamine, lidocaine, LC-MS/MS

## Abstract

Dried blood spot (DBS) sampling has emerged as a promising microsampling technique in biomedical and clinical research, offering advantages such as reduced invasiveness, minimal blood volume requirements, and enhanced analyte stability. Although well established in human medicine for neonatal screening and diagnostic applications, its potential in veterinary pharmacology remains underexplored. This study investigated the feasibility of using DBS samples to quantify anesthetic agents—ketamine and medetomidine in cats and lidocaine in horses—during routine surgical procedures at a veterinary teaching hospital. A standardized DBS collection protocol was developed, and LC-MS/MS methods were validated for the quantification of target analytes in both DBS and plasma samples. These methods were subsequently applied to real samples collected during anesthesia to conduct pharmacokinetic analyses. Comparative evaluations, including Bland–Altman analysis, assessed the suitability of DBS samples for pharmacokinetic studies in veterinary medicine. Preliminary results indicated satisfactory agreement for medetomidine, meeting EMA guidelines, with 75.6% of mean values falling within ±20% of paired measurements. Results for ketamine (46.9%) were promising but require further optimization, while those for lidocaine (21.4%) highlighted the need for additional investigation. These findings underscore the potential of DBS sampling as a minimally invasive alternative for pharmacokinetic studies in veterinary medicine, particularly for medetomidine, while identifying areas for further methodological refinement. Future research should optimize DBS techniques and expand their application to other drugs and species, broadening their impact on veterinary pharmacology.

## Simple Summary

Dried blood spot (DBS) sampling is a microsampling technique that involves collecting small volumes of blood on absorbent paper for later analysis. It offers several advantages, including minimal invasiveness, reduced blood volume requirements, and enhanced analyte stability. While widely used in human medicine for neonatal screening, diagnostics, pharmacokinetics, forensics, and infectious disease surveillance, its application in veterinary medicine remains limited. However, DBS sampling holds great potential in veterinary pharmacokinetic research by minimizing animal discomfort and simplifying sample handling. This study investigated the feasibility of using DBS sampling to quantify ketamine, medetomidine, and lidocaine levels in cats and horses undergoing surgery. The primary objectives were to develop a standardized DBS collection protocol, optimize LC-MS/MS analytical methods, and compare DBS with plasma samples.

## 1. Introduction

Currently, dried blood spot (DBS) sampling is recognized as a versatile microsampling technique in which small volumes of blood are collected on absorbent paper and dried for subsequent laboratory analysis. This method offers significant advantages over traditional approaches, including reduced invasiveness, minimal blood volume requirements, and improved analyte stability during storage and transport [1,2]. Furthermore, its cost-effective logistics favor the increasing adoption of DBS sampling across diverse fields of biomedical and clinical science [3,4,5,6].

In human medicine, DBS sampling is widely used for neonatal screening to detect rare genetic and metabolic disorders [6,7,8,9]. It is also employed in diagnostics, toxicokinetic and pharmacokinetic studies, clinical pharmacology, forensic science, and doping analysis [10,11,12,13,14,15]. Additional applications of DBS include infectious disease surveillance and therapeutic drug monitoring [13,16,17].

Despite being a well-established sampling method in human medicine, the use of DBS in veterinary medicine remains limited, providing scope for further exploration. Current research on the application of DBS in veterinary medicine predominantly focuses on virology, immunology, pharmacology, and toxicology [18,19,20,21,22,23,24,25,26,27,28].

However, in veterinary pharmacology, DBS offers a promising, minimally invasive alternative to plasma for pharmacokinetic research [10], reducing animal discomfort and addressing ethical considerations, thereby facilitating the approval of study protocols. While plasma and serum are traditionally considered the gold standard for pharmacokinetic studies, DBS samples offers a viable alternative that simplifies sample handling and minimizes logistical challenges. By overcoming these barriers, DBS sampling may expand research opportunities and improve the feasibility of pharmacokinetic investigations in veterinary settings.

This study aimed to assess the feasibility of using DBS samples for the quantification of three anesthetic agents, ketamine, medetomidine, and lidocaine, in cats and horses undergoing routine surgical procedures at our veterinary teaching hospital. In this exploratory study, our goal was to develop a standardized DBS collection protocol, optimize liquid chromatography-coupled tandem mass spectrometry (LC-MS/MS) methods for both DBS and plasma samples, and perform comparative analyses to evaluate the applicability of this technique for pharmacokinetic studies. The preliminary findings of this investigation may provide valuable insights into the feasibility and reliability of DBS as a microsampling approach for veterinary applications, offering insights into how this method can be improved for this particular context.

## 2. Materials and Methods

### 2.1. Chemicals, Reagents, and Materials

Analytical standards of medetomidine, medetomidine-d4, ketamine, ketamine-d4, lidocaine, and lidocaine-d10 were purchased from Toronto Research Chemicals (Toronto, ON, Canada). Acetonitrile, methanol, and formic acid (all of LC-MS grade), as well as ethyl acetate and dichloromethane, were obtained from Merck (Milan, Italy). Ultrapure water was freshly produced in-house using the Sartorius, Arium^®^ Ultrapure Water Systems (Varedo, Italy). Whatman 903 Protein Saver Cards (Whatman, UK), purchased from Merck (Milano, Italy), were used for spotting the blood samples.

Drug-free blood collected from healthy cats and horses was used for method development and validation.

### 2.2. Stock Solutions and Working Solutions

Each pure compound was prepared at a concentration of 1000 μg/mL by dissolving 10 mg in 10 mL of solvent—acetonitrile for ketamine and methanol for medetomidine—in precise volumetric flasks. A 1000 μg/mL stock solution of medetomidine-d₃ was similarly prepared by dissolving 10 mg of the labeled compound in 10 mL of methanol. A 10 μg/mL solution of ketamine-d4 in methanol was obtained by a 10-fold dilution of its 100 μg/mL stock solution in acetonitrile. The lidocaine stock solution (100 μg/mL) was prepared by diluting 1 mL of a 1000 μg/mL solution with 9 mL of methanol. Similarly, the lidocaine-d_10_ stock solution (10 μg/mL) was prepared by a 10-fold dilution of the 100 μg/mL solution. All stock solutions were stored at −20 °C in the dark.

Working solutions used for spiking calibrators and quality control (QC) samples, as well as those of internal standards, were freshly prepared by serial dilution on the day of analysis.

### 2.3. Investigated Drugs and Study Designs Employed

#### 2.3.1. Ketamine

The study involving ketamine was approved by the Animal Welfare Committee of the University of Bologna (Protocol No. 294336, dated 4 December 2020). This study enrolled seven male cats aged between three and four years, presented to the veterinary teaching hospital for castration procedures and treated with 20 µg/kg of medetomidine and 10 mg/kg of ketamine, both administered intramuscularly, to achieve sedation and muscle relaxation. Blood samples were collected at 10, 15, 20, 30, 45, and 60 min after drug administration. Venous blood collection via catheter and DBS sampling from the auricular pinna were performed simultaneously, as described in Section 2.4. A total of 38 plasma and 38 DBS samples were obtained. For three subjects, DBS samples at the two final time points could not be collected; therefore, the corresponding plasma samples were also excluded from the evaluations.

#### 2.3.2. Medetomidine

The medetomidine study, approved by the Animal Welfare Committee of the University of Bologna (Protocol No. 211643, dated 18 September 2019), involved seven male cats serving as semen donors. These cats were sedated with an intramuscular dose of 130 µg/kg of medetomidine administered into the area between the semitendinosus and semimembranosus muscles. Blood samples for plasma medetomidine analysis were collected at 10, 15, 20, 30, 45, 60, 75, 90, and 120 min from a cephalic catheter (*n* = 56), with simultaneous DBS sampling (*n* = 48) from the auricular pinna. Only the time points available for both matrices (*n* = 48) were included in the statistical analysis.

#### 2.3.3. Lidocaine

Six healthy horses (three geldings and three mares), aged between 1 and 11 years and classified as ASA Class ≤ 2 according to the American Society of Anesthesiologists (ASA) classification, were included in the study. The horses were admitted to the university teaching hospital for elective surgical procedures. The study was approved by the Animal Welfare Committee of the University of Bologna (Protocol No. 56507, dated 2 March 2023). The participants were initially subjected to general anesthesia (the detailed protocol is reported in Supplementary Materials Section S1). After confirming the adequacy of the anesthetic plan by assessing key reflexes and muscle relaxation, lidocaine infusion was initiated at a rate of 0.05 mg/kg/min using a syringe pump (Agilia Injectomat, Fresenius Kabi Italia, Verona, Italy). The blood and DBS sampling procedure was carried out at 5, 10, 30, 30, 45, and 60 min during lidocaine infusion. After 60 min, the lidocaine infusion was stopped, and subsequent samples were collected at 5, 10, 30, and 60 min after the interruption. It was not possible to collect samples at all time points for all patients; therefore, the total number of plasma and DBS samples was 54 each.

### 2.4. Sample Collection

#### 2.4.1. Plasma

Blood (0.5 mL) samples collected from the cephalic catheter in cats and from the jugular vein in horses were drawn and deposited into tubes containing EDTA. From each tube, an aliquot was taken using a capillary to estimate hematocrit (Hct) levels via packed cell volume (PCV). The remaining blood was then centrifuged at 4 °C at 2000× *g* for 10 min to obtain plasma for target analyte quantification and stored at −20 °C until the LC-MS/MS analysis.

Immediately after each blood draw, the catheters were flushed with saline solution (NaCl 0.9%) to prevent blood coagulation within the catheter.

#### 2.4.2. DBS

For all three drugs under investigation, DBS samples were collected from the auricular pinna (left or right side, chosen randomly) simultaneously with the blood collection using the procedure described below. First, the site was cleaned with gauze soaked in saline solution (NaCl 0.9%). Then, a small incision was made on the ear skin using a 22G needle (Microlance, Becton Dickinson S.A., Italia, Milan, Italy) to produce a drop of blood. In cases where the patient exhibited vasoconstriction, an alcohol wipe was applied to induce vasodilation, followed by a second cleaning with saline solution (NaCl 0.9%) to prevent any interference with sample analysis. Gentle massaging of the area was performed to enhance perfusion, ensuring an adequate blood flow and producing a drop of sufficient size.

The first drop of blood was discarded to avoid contamination with intracellular or interstitial fluids. The subsequent drop was collected using a calibrated micropipette to obtain a precise volume of 20 µL. This sample was then deposited onto Whatman 903 filter paper, ensuring placement at the center of the designated circle and aiming for a uniform distribution of the blood.

**Figure 1:**
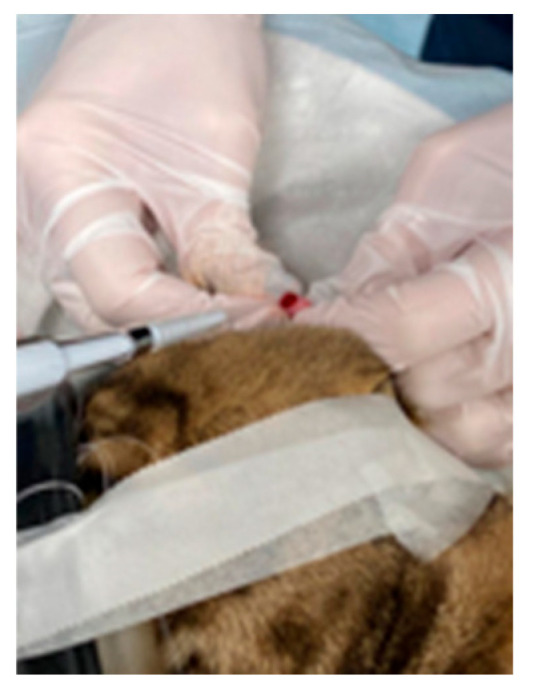
The blood samples were taken by pricking the cat’s ear with a needle. A 20 µL volume was collected using a P20 Gilson Pipette and deposited onto Whatman Protein Saver 903 paper.

Dried blood spot samples were left to dry at room temperature, away from heat sources, with care taken to prevent contamination. After the final collection, all samples were allowed to dry for 2 h and then stored in plastic bags at room temperature, protected from light, for up to 24 h before analysis. A representative image of the DBS sampling procedure is shown in Figure 1.

### 2.5. Sample Preparation

#### 2.5.1. Plasma Extraction

For ketamine extraction, 200 µL of feline plasma was transferred into a 0.5 mL Eppendorf microtube containing 20 μL of water and 200 μL of internal standard ketamine-d4 solution in acetonitrile (1 μg/mL). The microtubes were vortexed for 30 s and then centrifuged at 21,000× *g* for 10 min at 20 °C. Following centrifugation, 100 μL of the supernatant was transferred into a chromatography vial, diluted with 200 μL of water containing 0.1% formic acid, and subsequently injected into the LC-MS/MS system.

Medetomidine in cat plasma was extracted following the procedure described here [29]. Briefly, 200 µL of feline plasma was spiked with 20 µL of medetomidine-d4 internal standard (0.5 µg/mL), mixed with 1 mL of ethyl acetate, and centrifuged at 21,000× *g* for 15 min at 4 °C. The supernatant was evaporated under a gentle nitrogen stream at 40 °C, and the resulting dry residue was reconstituted in 300 µL of mobile phase (40:60, *v*/*v*, water with 0.1% formic acid and acetonitrile) and injected in the analytical system.

For lidocaine extraction, 200 µL of equine plasma was transferred into an Eppendorf microtube containing 400 μL of an 70:30 (*v*/*v*) acetonitrile/zinc sulfate (0.1 M) aqueous solution, along with 20 μL of lidocaine-d10 at a concentration of 0.5 μg/mL in water. The mixture was vortexed for 30 s and centrifuged at 21,000× *g* for 10 min at 20 °C. Following centrifugation, 50 μL of the supernatant was transferred into a chromatography vial and diluted with 200 μL of water adjusted to pH with 0.1% formic acid. The final prepared sample was injected into the LC-MS/MS system for analysis.

#### 2.5.2. DBS Extraction

For each dried blood spot (dried for a minimum of 24 h at room temperature), the entire section of the filter (Whatman Protein Saver 903) containing the whole blood spot, equivalent to 20 μL, was excised and transferred into a 1.5 mL Eppendorf microtube. The cut spot was combined with 300 μL of water and 20 μL of an aqueous solution of the respective internal standard (ketamine-d4 at a concentration of 1 μg/mL, medetomidine-d4 at a concentration of 0.1 μg/mL, or lidocaine-d10 at a concentration of 0.5 μg/mL) and vortexed for 30 s. Subsequently, 700 μL organic solvent (acetonitrile for ketamine and lidocaine and methanol containing 0.1% of formic acid for medetomidine) was added, followed by an additional 30 s vortex mixing. Samples were then placed in an ultrasonic bath for 1 h. Following sonication, samples were centrifuged at 21,000× *g* for 10 min at 20 °C.

For medetomidine, 850 µL of the supernatant was collected and evaporated to dryness under nitrogen at 45 °C. The dry residue was subsequently reconstituted in 200 µL of mobile phase (H20 + 0.1% AF:ACN 40:60 *v*/*v*) and transferred to an LC vial.

For the other compounds, the supernatants were diluted 2× (for lidocaine) and 7× (for ketamine) in an LC vial containing ultrapure water acidified with 0.1% formic acid and injected into the LC-MS/MS system.

### 2.6. Drug Quantification

Drug quantification was performed using a liquid chromatography tandem mass spectrometry (LC–MS/MS) approach. The LC system consisted of a Waters Acquity UPLC^®^ binary pump (Waters, Milford, MA, USA) equipped with an ACQUITY UPLC BEH C18 column (1.7 μm, 2.1 × 50 mm) and a corresponding precolumn. The column temperature was maintained at 40 °C for the analysis of medetomidine and ketamine and at 35 °C for lidocaine.

For the three compounds, water with 0.1% formic acid (A) and acetonitrile (B) were used as mobile phases under various programmed conditions, as shown in Table 1. Extracted samples were maintained at 20 °C in the autosampler, and 10 μL aliquots from each vial were injected into the analytical system.

The LC was interfaced with a Waters XEVO TQ-S Micro triple quadrupole mass spectrometer (Waters, Milford, MA, USA), operating in positive electrospray ionization (ESI+) and in multiple reaction monitoring (MRM) mode. The capillary voltage was set to 0.5 kV for medetomidine, 3.0 kV for ketamine, and 0.75 kV for lidocaine, while the source and desolvation temperatures were set 150 °C and 600 °C, respectively, for all analytes. The cone gas was set to 50 L/h and desolvation gas to 900 L/h; argon was used as a collision gas. The analyte-dependent MS/MS parameters were optimized by simultaneously infusing the LC mobile phase and standard solutions of each analyte into the mass spectrometer. The most abundant transitions for the three analytes and their internal standards were identified and are reported in Table 2, along with their corresponding cone voltage and collision energy values. Data acquisition and analysis was performed using MassLynx 4.2 software (Waters, Milford, MA, USA).

In this exploratory phase of the study, the developed analytical methods for quantifying the studied drugs in plasma and DBS were preliminarily performed in accordance with the European Medicine Agency ICH M10 guidelines [30]. For each study, the considered parameters included selectivity, calibration range, lower limit of quantification (LLOQ), accuracy, precision (coefficient of variation, CV%), and carry-over. Calibrators and quality control (QC) samples were prepared with both plasma and whole blood by spiking 200 μL aliquots of each matrix with 10 μL of working solutions containing the target analyte at the corresponding concentrations. To avoid hemolysis caused by organic solvents, working solutions intended for whole blood were prepared in water. After spiking, the samples were gently mixed and allowed to equilibrate for 30 min at room temperature. Subsequently, 20 μL of the spiked blood was aliquoted and applied to the Whatman card for the preparation of the calibration curve for DBS. Calibration curves were constructed using the linear equation *y* = *ax* + *b*, with a weighting factor of 1/*x* applied to account for heteroscedasticity across the concentration range.

Briefly, the LLOQ was defined as the lowest concentration measured in the samples that could be detected with a signal-to-noise (S/N) ratio ≥ 10 and acceptable accuracy (within ±20%) and precision (CV < 20%) after the injection of four replicates. Accuracy, expressed as the relative difference between measured value and expected concentration, was evaluated at each QC level and considered acceptable if within ±15% of the nominal concentration. Similarly, precision, defined as the coefficient of variation (CV%) among repeated individual measures, had to be <15% for each QC level.

The calibrators, LLOQ, and QC samples (in bold) for each compound in plasma and DBS are reported in Table 3.

Carry-over contamination was evaluated by analyzing six drug-free plasma or DBS samples after the injection of the highest calibrators. The analytical response in the blank samples had to be below 20% of the LLOQ.

### 2.7. Statistical Analysis

Statistical analyses were conducted using MedCalc^®^ version 23.0.9 (MedCalc Software, Ostend, Belgium). Outliers were identified by assessing the ratios of paired plasma and DBS concentrations for each analyte before performing Deming regression; any ratio falling outside 1.5 times the interquartile range was considered an outlier [31]. Additionally, samples with concentrations below the LLOQ were excluded from the analysis [32]. The correlation between analyte concentrations in plasma and DBS was evaluated using Deming regression, which accounts for measurement errors in both variables [31]. These errors were derived from the inter-assay coefficient of variation obtained during assay validation for both plasma and DBS samples. The extent of correlation was determined using the Pearson correlation coefficient (r). Predicted plasma concentrations were calculated from observed DBS concentrations using the Deming regression equation: predicted plasma = *m* + *b* × DBS, where *m* represents the intercept and *b* the slope of the regression line.

The agreement between observed and predicted plasma concentrations was assessed following the European Medicines Agency ICH M10 guidelines, which require that at least 67% of the samples show a difference of less than 20% between observed and predicted plasma concentrations [30]. Finally, the differences between observed and predicted plasma concentrations were visualized using Bland–Altman plots.

Bland–Altman plots were generated after filtering out values below the LLOQ. Additionally, separate plots were created by excluding values below 10 ng/mL for medetomidine and 500 ng/mL for ketamine and lidocaine.

## 3. Results

Once the chromatographic conditions for each compound and its internal standard were optimized, their retention times (1.35 min for medetomidine, 1.89 min for ketamine, and 1.48 min for lidocaine) were determined by injecting individual pure solutions at a concentration of 100 ng/mL. Figure S1, included in the Supplementary Materials, shows representative chromatograms of each target analyte in both matrices. The selectivity of the method was determined by analyzing blank plasma and DBS samples, confirmed by the absence of chromatographic signals at the same elution time as the target analytes.

For medetomidine, the LLOQ was 1 ng/mL in plasma and DBS. For ketamine, the LLOQ was 250 ng/mL in both plasma and DBS, and for lidocaine, the LLOQ was 100 ng/mL in plasma and 250 ng/mL in DBS. Calibration curves in both plasma and DBS, prepared on separate testing days, consistently exhibited a coefficient of determination (R^2^) ≥ 0.99. Furthermore, calibrators always fell within ±15% of the expected value, demonstrating the linearity of the method across the validated concentration ranges.

For ketamine, calibration curves covered a range of 250–5000 ng/mL in both plasma and whole blood; for lidocaine, a range of 100–5000 ng/mL for plasma and 250–10,000 ng/mL in whole blood; and for medetomidine, a range of 1–200 ng/mL for both matrices. For all the analyses, accuracy at each QC level was within ±15% of the nominal concentration, and precision was less than 15% at each QC level as per EMA criteria. The absence of carry-over contamination was confirmed by analyzing drug-free plasma and DBS samples following the injection of the highest calibrators. In the blank samples, the response was found to be lower than 20%, specifically around 8% for ketamine, 3% for medetomidine, and 12% for lidocaine.

The concentration–time curves obtained from plasma and DBS samples for the three analytes are presented in Figure S2 of the Supplementary Materials.

**Figure 2:**
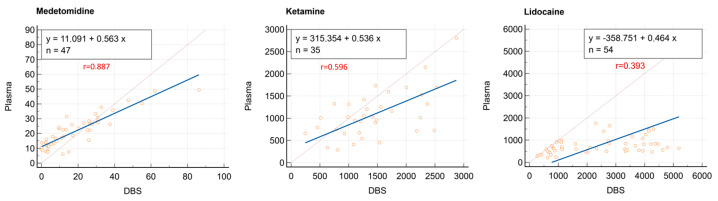
Deming regression for medetomidine (*n* = 47), ketamine (*n* = 35), and lidocaine (*n* = 54). Pearson’s correlation coefficient is denoted as *r*. The blue line represents the line of identity (X = Y), the red line depicts the Deming regression line accounting for measurement error in both variables, and the red circles correspond to individual paired observations.

The paired plasma and DBS concentration ratios with corresponding outliers (one for medetomidine, four for ketamine, and two for lidocaine), are shown in Figure S3 and reported as Supplementary Materials. Figure 2 also presents the Deming regressions with Pearson correlation coefficients (r) for medetomidine, ketamine, and lidocaine. The 95% confidence intervals (CIs) for the slope were 0.8059–0.9362 for medetomidine, 0.3292–0.7759 for ketamine, and 0.1405–0.5982 for lidocaine. DBS concentrations were corrected for bias using Deming regression equations to derive corresponding plasma concentrations.

Agreement between derived and observed plasma values was assessed using Bland–Altman plots.

**Figure 3:**
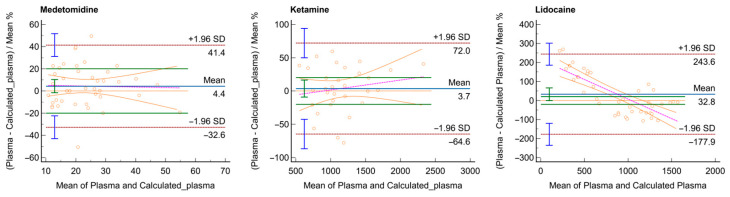
Bland–Altman plots evaluating the agreement between the derived plasma and observed plasma concentrations for medetomidine, ketamine, and lidocaine. The solid green lines, positioned on either side of the solid blue line (representing the mean percentage error), indicate the 20% acceptable bias range. The pink line represents the mean difference (or bias) between the two methods, showing the average difference between the two sets of measurements. The orange lines represent the lower limits of agreement, calculated as the mean difference minus 1.96 times the standard deviation, defining the lower boundary for 95% of the differences. The blue lines show the variability of individual data points, including the standard deviation at each measurement point. Individual paired differences are shown as small circles, and the brown dotted lines represent the limits of agreement, defined as the mean difference ± 1.96 standard deviations.

The results of the first set of Bland–Altman plots did not indicate good agreement for ketamine and lidocaine (plots are reported in Figure S4 as Supplementary Materials). As a result, further plots were generated by filtering out values below 10 ng/mL for medetomidine and below 500 ng/mL for ketamine and lidocaine. The Bland–Altman plots obtained using the new cut-off for analytes reported in Figure 3 show a limited proportional bias for medetomidine (A) and ketamine (B). The lidocaine plot (C) shows a clear proportional bias, as indicated by the downward trend in the plot with increasing mean values. This indicates that the differences between the measurements systematically decrease as the mean of the measurements increases. The percentage of samples of each drug where the difference of the two measurements falls within ±20% of the mean values of the two measurements is 75.6% for medetomidine, 46.9% for ketamine, and 21.4% for lidocaine.

## 4. Discussion

Although DBS microsampling is widely used across various fields due to its well-known advantages [2,12,15,33], it presents challenges such as optimizing sample collection procedures; addressing analytical issues like spotting volume, hematocrit, and spot inhomogeneity; and a lack of specific regulatory guidelines for assay validation [34,35,36]. Various studies offer strategies to address these challenges and guide DBS protocol development [4,5,37,38,39,40,41,42].

However, in veterinary medicine, the use of DBS sampling remains limited, especially for pharmacokinetic studies in companion animals. While previous research has explored DBS for biobanking and metabolomics [24] and for use in pharmacokinetic studies on laboratory animals such as rats [43,44], to our knowledge, no studies have yet applied this technique to real-world veterinary pharmacokinetic settings. Therefore, this study focused on evaluating the suitability of this microsampling technique for pharmacokinetic studies. For ethical reasons, we used patient-derived data by collecting samples from patients undergoing surgery at our veterinary teaching hospital. Specifically, we compared DBS concentrations of the anesthetic agents medetomidine, ketamine, and lidocaine with plasma levels to better understand the correlation between these two sampling methods.

Crucial to this study was the standardization of the DBS collection protocol in cats for medetomidine and ketamine studies and in horses for the lidocaine study. The developed strategy involved collecting a fixed volume of whole blood, corresponding to 20 µL, using a calibrated laboratory pipette, which was then deposited onto the filter card. After drying for the necessary time, the entire spot was cut and extracted. This strategy was optimized to minimize or reduce potential hematocrit effects. To optimize the DBS extraction procedure for all the considered drugs, various organic solvents, including acetonitrile, methanol, ethyl acetate, and dichloromethane, were tested in different proportions with the addition of varying percentages of formic acid. The best results for ketamine and medetomidine were achieved by first extracting the spot with 30% water and then adding the organic solvent. For medetomidine extraction from DBS, as well as plasma, it was necessary to concentrate the drug by drying at 45 °C under nitrogen, followed by reconstitution in the mobile phase. The plasma extraction procedures for ketamine and medetomidine were adopted from our previous studies [29,45] with slight modifications.

The optimal chromatographic results for lidocaine extraction from plasma were achieved using a mixture of acetonitrile and 0.1 M zinc sulfate in water at a 70:30 (*v*/*v*) ratio. For the LC-MS/MS method optimization, various tests were performed using different combinations of mobile phases, gradients, and analytical columns. The best results in terms of peak shape and analytical response for the three target analytes and their internal standards were obtained with a BEH C18 (1.7 μm, 2.1 × 50 mm) column under different gradient conditions (reported in Table 1).

In this study, the DBS microsampling technique and LC-MS/MS methods were applied to three distinct groups of animals undergoing anesthesia with different protocols. The primary objective was to investigate the quantification of ketamine and medetomidine in cats, as well as lidocaine in horses, using DBS samples obtained from patients and to compare the results with those obtained from plasma samples. This work aimed to gain further insights into the behavior of these drugs in dried matrices and to assess the potential of DBS as a viable technique for pharmacokinetic investigations in veterinary medicine.

Preliminary results from the experiments and statistical analyses showed satisfactory outcomes for medetomidine, where 75.6% of the samples exhibited a difference of less than 20% between observed and predicted plasma concentrations, which is in agreement with ICH M10 guidelines [30]. On the other hand, ketamine and lidocaine did not meet this criterion. Given the differences in sample collection sites for plasma and DBS samples, as well as potential physiological variations, additional thresholds for agreement were explored. Specifically, the variability in agreement between observed and predicted plasma concentrations was assessed using broader thresholds of ±30% and ±40%. This approach provided a more comprehensive evaluation of the method’s applicability considering various physiological differences. When a ±40% difference was applied, 62.9% of the samples from the ketamine study met the threshold. However, for lidocaine investigations, the results remained unsatisfactory, even when considering differences of ±30% and ±40%. In this context, this preliminary research provided valuable insights into the compounds for which DBS could be a suitable microsampling technique, such as medetomidine and, if considering a ±40% difference, ketamine. These findings underscore the need for further investigations into the suitability of DBS for quantifying lidocaine and ketamine. In humans, lidocaine exhibits concentration-dependent binding to plasma proteins, particularly α1-acid glycoprotein (AAG) [46,47]. As lidocaine concentrations increase, AAG sites become saturated, resulting in a greater proportion of free (unbound) drug. Because free lidocaine partitions differently between plasma and blood cells, its distribution into whole blood increases at higher total concentrations. Consequently, DBS samples may overestimate plasma concentrations at high lidocaine levels and underestimate them at lower levels, as reflected in the trend observed in the Bland–Altman plot. Additionally, species-specific hematological differences may influence DBS performance. According to reference intervals established by our clinical pathology service—following international guidelines [48] and based on data from 120 animals per species—healthy horses generally exhibit higher hematocrit levels (32–52%) than healthy cats (32–48%). We hypothesize that this elevated hematocrit may limit blood spread on filter paper, leading to a denser matrix and potentially uneven analyte distribution. Although punching the entire spot mitigates sampling bias, the increased viscosity and stronger adhesion of equine blood to the paper substrate may still compromise extraction efficiency. Therefore, in future studies, it will be essential to include hematocrit assessment at each sampling time point to better account for its potential impact on sample quality and analyte distribution.

To the best of our knowledge, no studies have described the red blood cell partitioning of medetomidine and lidocaine in either cats or horses. In contrast, the distributions of other agents—such as the alpha-2 agonist romifidine and the dissociative anesthetic ketamine—have been previously investigated in horses [49,50]. This highlights the need for further research to characterize the disposition of medetomidine and lidocaine across different species and compartments. Another limitation of this study is the lack of external validation for the DBS-to-plasma concentration conversion. While Deming regression and Bland–Altman analysis were performed on patient samples, the model was not tested on an independent validation set. Incorporating such validation in future research would help differentiate analytical variability from biological or matrix-related effects.

The site of blood collection may also influence drug distribution. In large animals, such as horses, sampling from the jugular vein versus the auricular pina—even when performed simultaneously—may result in differences in analyte concentrations. To address this issue, future research can benefit from a study design in which blood for both plasma and DBS is collected from the same site. Obtaining a DBS from the jugular vein would allow for a more direct comparison with plasma samples collected from the same site. Furthermore, it may be interesting to develop a method for quantifying lidocaine in red blood cells to better understand the behavior of this drug in equine whole blood, red blood cells, and plasma. Findings from these additional evaluations may provide valuable insights into the feasibility and reliability of DBS as a microsampling technique for lidocaine quantification in research studies on horses.

## 5. Conclusions

In conclusion, this study explored the potential of dried blood spots as a viable and minimally invasive alternative to plasma for pharmacokinetic research in veterinary applications involving anesthetics. The validated LC-MS/MS methods for both DBS and plasma samples demonstrated effective quantification of ketamine and medetomidine in cats, as well as lidocaine in horses. The preliminary results were satisfactory for medetomidine, promising for ketamine, and highlighted the need for further investigation regarding lidocaine. The standardized and optimized DBS collection protocol established in this study provides a robust foundation for future research, enhancing the applicability of this microsampling technique in pharmacological studies in both small and large animals. These findings pave the way for broader investigations and the potential adoption of DBS sampling in veterinary pharmacology, offering an efficient and innovative approach to expand our understanding of drug pharmacokinetics in diverse animal populations.

## Figures and Tables

**Table 1:** Chromatographic gradients for ketamine, medetomidine, and lidocaine.

Ketamine			
Time (min)	Flow (mL/min)	% A	% B
0.00	0.300	90	10
1.00	0.300	90	10
1.80	0.300	5	95
3.00	0.300	5	95
3.50	0.300	90	10
4.00	0.300	90	10
**Medetomidine**			
**Time (min)**	**Flow (mL/min)**	**% A**	**% B**
0.00	0.350	40	60
1.00	0.350	5	95
2.70	0.350	5	95
3.00	0.350	40	60
3.50	0.350	40	60
**Lidocaine**			
**Time (min)**	**Flow (mL/min)**	**% A**	**% B**
0.00	0.400	65	35
0.20	0.400	65	35
0.80	0.400	5	95
2.50	0.400	5	95
3.00	0.400	65	35
4.00	0.400	65	35

**Table 2:** Optimized MS/MS transitions, cone voltage, and collision energy for medetomidine, ketamine, lidocaine, and their internal standards.

Analyte	MRM Transition (*m*/*z*)	Cone Voltage (V)	Collision Energy (eV)
Ketamine	238.1 > 124.9	20	26
Ketamine-d4	242.0 > 129.0	20	26
Medetomidine	201.1> 94.9	28	18
Medetomidine-d4	204.1 > 97.9	28	18
Lidocaine	235.1 > 85.9	30	17
Lidocaine-d10	245.1 > 95.9	30	18

**Table 3:** Concentrations of the calibrators (*n* = 5 for ketamine; *n* = 6 for medetomidine and lidocaine) and three QC levels prepared for each target analyte in DBS and plasma.

	Plasma			DBS		
Level	Ketamine (ng/mL)	Medetomidine (ng/mL)	Lidocaine (ng/mL)	Ketamine (ng/mL)	Medetomidine (ng/mL)	Lidocaine (ng/mL)
1 (+QC)	250	1	100	250	1	250
2	500	5	250	500	5	500
3 (+QC)	1000	20	500	1000	20	1000
4	2500	50	1000	2500	50	2500
5 (+QC)	5000	100	2500	5000	100	5000
6	-	200	5000	-	200	10,000

## Data Availability

The original contributions presented in this study are included in the article and in the Supplementary Materials. Further inquiries can be directed to the corresponding author.

## Supplementary Materials

The following supporting information can be downloaded at: https://www.mdpi.com/article/10.3390/vetsci12050488/s1. Section S1. Detailed protocol of general anesthesia of the 6 horses enrolled in the study. Figure S1. Representative chromatograms of target analytes obtained from plasma (left) and DBS (right) samples collected 30 min post-administration. Figure S2. Concentration–time profiles of medetomidine, ketamine and lidocaine in plasma and DBS. Figure S3. Box-and-whisker plots of plasma to DBS concentration ratios for medetomidine, ketamine, and lidocaine., Figure S4. Bland-Altman plots evaluating the agreement between the derived plasma and observed plasma concentrations for medetomidine, ketamine, and lidocaine.
