# Supplementary material for: The Suitability of Dried Blood Spot Sampling for Pharmacokinetic Studies in Veterinary Medicine
Source: Vet Sci. 2025 May 18;12(5):488. doi: 10.3390/vetsci12050488 (PMC12115528; doi:10.3390/vetsci12050488)

Section S1. Detailed protocol of general anesthesia of the 6 horses enrolled in the study.

The horses included in the study were admitted at Bologna Veterinary Teaching Hospital (VTH) to be undergone to elective surgical procedure. The inclusion criteria were:

- physical status American Society of Anaesthesiologists 1 or 2 based on a physical examination;
- age ≥ 1 year;
- non-food-producing horses.

After an aseptic preparation of the field, a right jugular catheter 14G (Intranule, VYGON) and left jugular catheter 18G were placed following desensitization of the insertion site with 1 mL of lidocaine 2% (lidocaine HCL 2%; Ecuphar italia s.r.l., Italia). The first catheter was used for administration of the anaesthesia drug and the second one for the withdrawal of the blood.

Each horse was sedated with intramuscular (IM) acepromazine (0.03 mg/kg; Prequillan; Fatro, Italy), intravenous (IV) romifidine (0.08–0.1 mg/kg; Sedivet; Boehringer Ingelheim, Belgium), and IV methadone (0.05 mg/kg; Semfortan; Dechra Pharmaceutics, Northwich, UK). The horses were then moved into a padded box, where the anaesthesia was induced with the aid of a gate by two operators: one controlling the head of the horse, and the anaesthetist controlling the gate. General anaesthesia was induced with an IV combination of diazepam (0.05 mg/kg IV; Ziapam; Ecuphar, Belgium) and ketamine (2.5 mg/kg IV; Nimatek; Dechra, Italia). Once the horse was recumbent, the trachea was intubated, and the animal was placed on a surgical table and connected to a large-animal circle system. The anaesthesia was maintained using isoflurane delivered in a mixture of air and oxygen (1:1 ratio), in combination with a romifidine infusion (0.04 mg/kg/h) and lidocaine (0.05 mg/kg/min).

Heart rate, mean arterial pression, FIO2, end-tidal CO_2, and partial end-tidal Isofurane were recorded every 5 minutes. Dobutamine up to 2 mcg/kg/min (Dobutamina; Bioindustria LIM, Italy), administered via an infusion pump (Agilia Injectomat, Fresenius Kabi, Italy), and Ringer's lactated solution 5 mL /kg/h (Ringer Lattato, ACME, Italy) were administered IV to maintain MAP > 70 mmHg. After the positioning of the monitoring, a 20G catheter was insert in facial vein to collect the blood from peripheral blood. A 1 mL sample of blood was collected in EDTA tube from the right jugular and the facial vein.

Figure S1. Representative chromatograms of target analytes obtained from plasma (left) and DBS (right) samples collected 30 min post-administration.

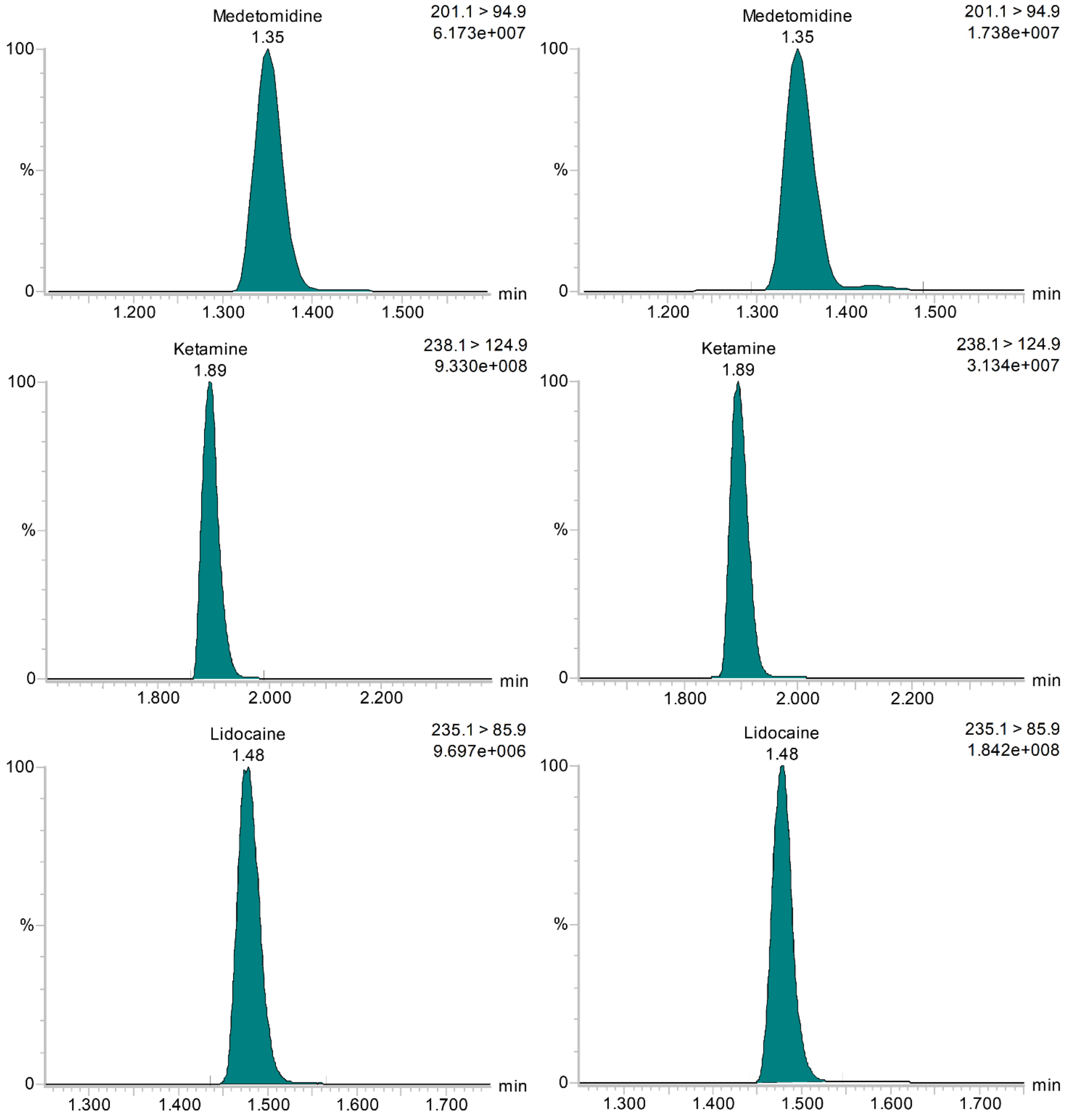

Figure S2. Concentration–time profiles of medetomidine, ketamine and lidocaine in plasma and DBS.

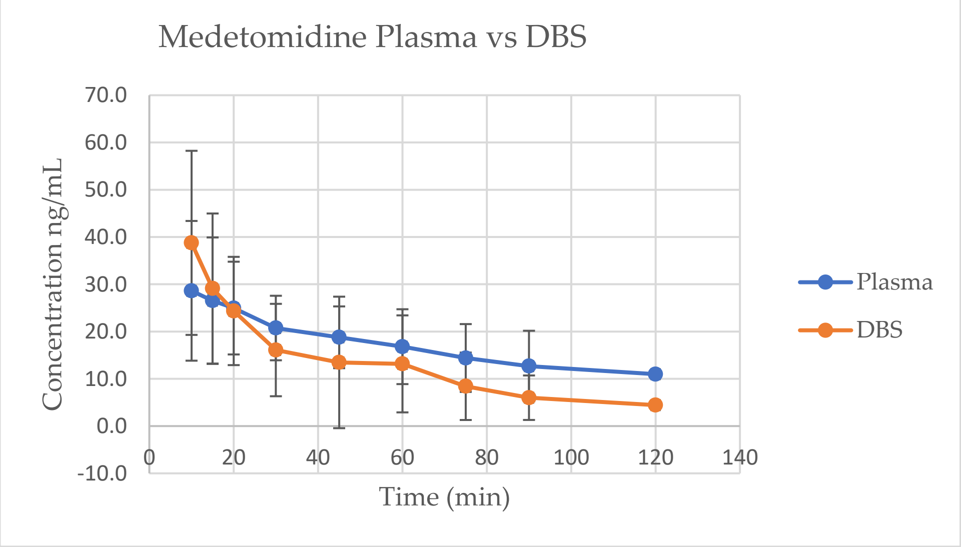

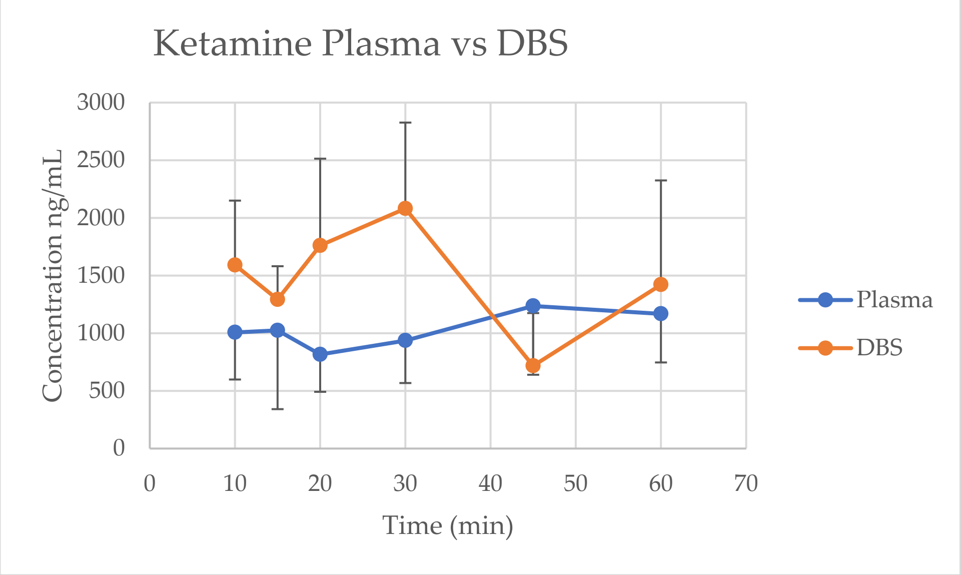

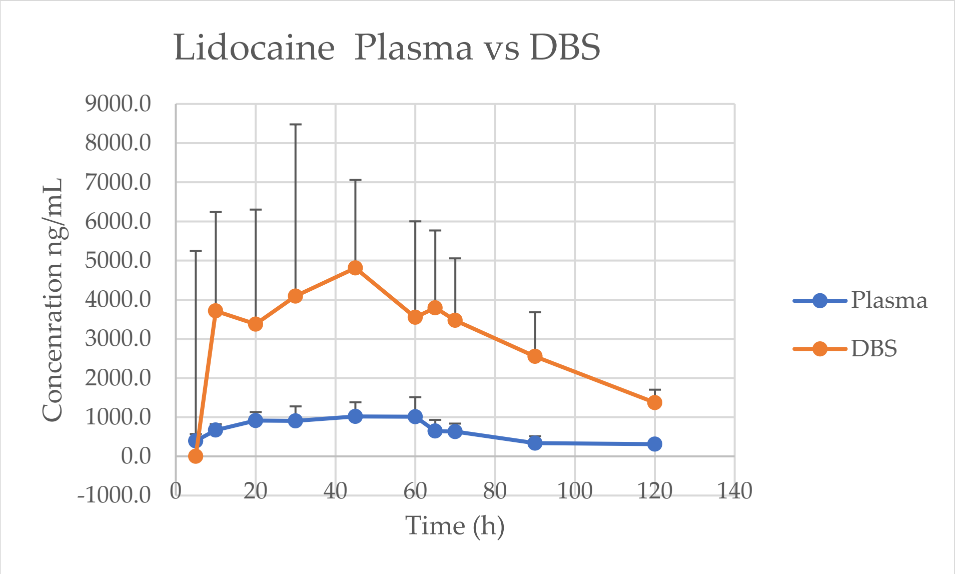

Figure S3. Box-and-whisker plots of plasma to DBS concentration ratios for medetomidine (A), ketamine (B), and lidocaine (C).

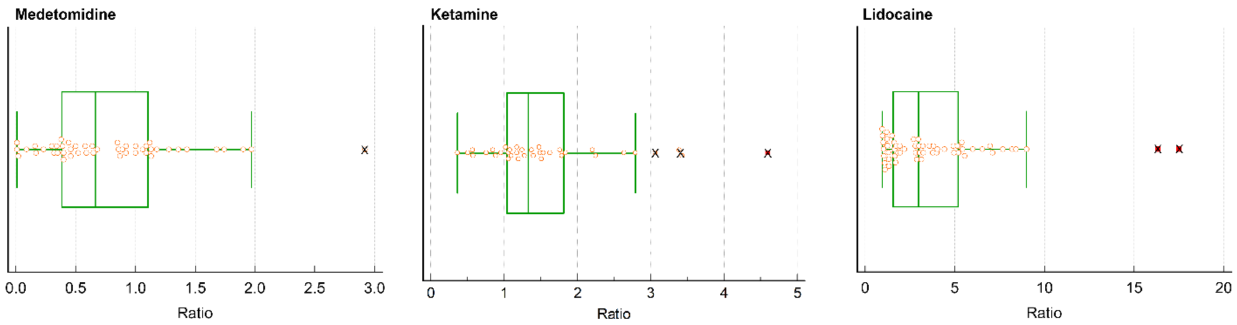

Figure S4. Bland-Altman plots evaluating the agreement between the derived plasma and observed plasma concentrations for medetomidine, ketamine, and lidocaine.

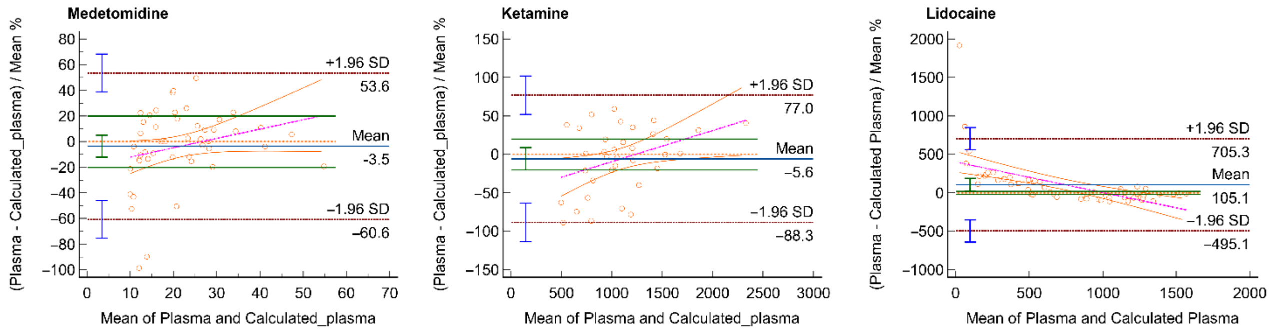

Supplement: Supplementary file 1 [file vetsci-12-00488-s001.zip › vetsci-3573732-supplementary.pdf]
